# Acceptability and use of an online health priorities self-identification tool for older adults: A qualitative investigation

**DOI:** 10.1016/j.pecinn.2023.100242

**Published:** 2023-12-08

**Authors:** Emily L. Mroz, Kizzy Hernandez-Bigos, Jessica Esterson, Eliza Kiwak, Aanand Naik, Mary E. Tinetti

**Affiliations:** a Section of Geriatrics, Department of Internal Medicine Yale School of Medicine, New Haven, CT, USA; b Institute on Aging, University of Texas Health Science Center, Houston, TX, USA; c Houston Center for Innovations in Quality, Effectiveness, and Safety, Michael E. DeBakey Veterans Affairs Medical Center, Houston, TX, USA; d Department of Chronic Disease Epidemiology, Yale School of Public Health, New Haven, CT, USA

**Keywords:** User-centered design, Patient priorities care, Geriatrics, Healthcare decision-making, My health priorities, Web-based tool

## Abstract

**Objective:**

To examine the use of a web-based, self-directed health priorities identification tool for older adults with multiple chronic conditions (MCCs).

**Methods:**

We recruited a gender- and racially-diverse, highly educated sample of older adults with MCCs to engage with our My Health Priorities tool, then complete a semi-structured interview. Thematic analysis was used to examine interview transcripts.

**Results:**

Twenty-one participants shared perspectives on the acceptability and use of the tool. Three themes (with eleven subthemes) were generated to describe: website *user experience feedback*, the *content of the health priorities identification process*, and the tool's *capacity to empower communication and decision making.*

**Conclusion:**

Participants found this tool acceptable and easy to use, describing a variety of benefits of the priorities self-identification process and offered suggestions for refinement and broader implementation. Older adults with limited internet navigation abilities or misconceptions about the self-directed process may benefit from clinicians clarifying the purpose of the process or initiating priorities-aligned discussions.

**Innovation:**

This novel tool can help older adults with MCCs define what matters most for their health and healthcare, informing a variety of health decisions. This tool may enable and motivate patients to lead health priorities decision-making discussions with clinicians and care partners.

## Highlights

• Older adults with multiple chronic conditions benefit from identifying health priorities.
• A web-based tool supported self-identification of health priorities.
• Users felt empowered to engage in priorities-aligned discussions with clinicians and family.
• Users identified structural and content refinements that may improve usability.

## 1 Background

The number of older adults experiencing multiple chronic conditions (MCCs) is increasing [1,2]. Healthcare offered to individuals with MCCs is often of unclear benefit, burdensome, and not focused on what matters to these individuals. One promising strategy to address these challenges is to move from *disease-centered* to *health priorities-directed* healthcare decision-making [3]. To support this move, a multistakeholder group developed Patient Priorities Care, which involves aligning care and treatments with patients' self-identified top priorities for their health and healthcare [[4], [5], [6]]. As part of this work, researchers developed a facilitator-supported patient priorities identification process for use in clinical settings [7,8]. This process increases priorities-concordant care, including reducing unwanted medications, in older adults with MCCs [9,10].

While facilitator-directed priorities identification was effective and remains important for patient care [7], many practice sites lack personnel, space, or time to facilitate guided priorities identification. Therefore, our study team designed a self-directed, web-based priorities identification tool, the My Health Priorities website (https://myhealthpriorities.org/) that could be completed virtually, in any setting. Such a tool nudges control towards the patient, allowing patients to identify their health goals without clinical time constraints, and potentially initiate goals-directed discussions in clinical settings [11]. We followed tenets of user-centered design [7,[12], [13], [14], [15]] when designing our tool. Completed steps included 1) characterizing the intended users and features (i.e., older adults with MCCs), 2) creating a prototype that addresses intended users' needs by adapting existing Patient Health Priorities Identification guides, and 3) conducting iterative cycles of feedback and refinement in conjunction with older adults and experts in older adult health and computer literacy. The result was a HIPAA compliant, interactive, user-informed website tailored to older adults with MCCs. This tool guides users through steps to identify specific, realistic, and actionable health goals based on their values, that is, what gives meaning, purpose, and joy in their current life; preferences for health care in the face of trade-offs (e.g., medications, healthcare visits); most bothersome health problem; and top health priority (i.e., the “One Thing” the person most wants to focus on). Users receive a printable, savable form based on their responses.

The current study is a qualitative examination of the acceptability and ease of use of the My Health Priorities self-identification tool in a sample representing end users (i.e., older adults with MCCs). The guiding research question was: *in what ways, and under what circumstances, is the My Health Priorities Self-Identification website acceptable and useful for guiding health priorities identification and discussions for older adults living with MCCs?*

## 2 Method

Methods and results are reported in accordance with the Consolidated Criteria for Reporting Qualitative Research. [16] Ethical approval for this study was conferred by the study team's institutional review board (HIC#: 2000029526).

### 2.1 Participants, recruitment, and enrollment

Recruitment strategies were continuously reviewed by the study team for purposive sampling of men and women from diverse cultural and geographical backgrounds. While our goal was to recruit a sample representative of potential users, our analysis was not intended to generate distinctions between individuals from different demographic backgrounds. Potential participants were identified through referral from collaborating organizations, word of mouth, and a national online research recruitment website (researchmatch.org). Eligibility criteria included: age 70 years or older, at least three health conditions, and saw at least two clinicians to manage conditions over the past year. Exclusion criteria were inability to provide informed consent, non-English-speaking, previous enrollment in Patient Health Priorities Identification studies, or no access to a computer. The interviewer ensured potential participants met these criteria before proceeding with enrollment. Participants received a $20 gift card following study completion.

### 2.2 Study design and procedures

Data collection occurred from July 2021 to February 2023. Two trained, experienced interviewers conducted screening and semi-structured interviews with participants via phone or video conferencing, depending on their preference. The interview facilitation questions were first pilot tested with four older adults. See Appendix A for the final interview guide.

Interested participants voluntarily contacted the research team by email or phone, as indicated by study flyers and recruitment announcements. After assessing eligibility, interviewers garnered participant informed consent, shared instructions for website use, and scheduled the study interview. All participants agreed to the website Terms of Use. Within two days of completing the website individually, participants completed the semi-structured study interview and provided demographic information. Audio-recorded interviewers were transcribed using a paid transcription service and checked by one interviewer for accuracy.

### 2.3 Data analysis

Data analysis was guided by a *codebook thematic analysis* approach following the *constant comparative method* [17], which guides iterative comparing of emergent concepts to identify themes describing a populations' experiences. Codebook analysis approaches emphasize efficient synthesis of the data to generate a codebook offering pragmatic takeaways [18]. Two coders with complementary backgrounds led thematic analysis. One (KHB) helped to develop the My Health Priorities Website, was familiar with the Patient Health Priorities Identification processes, recruited participants, and led most interviews. The other (ELM) had expertise in qualitative methods and patient health decision-making and goals-directed care but had not previously been involved with the Patient Priorities Care research team. The study team's approaches for upholding qualitative rigor and trustworthiness are further described in Appendix B.

The analysis process involved familiarization, open coding, initial theme generation, and comparison of candidate themes and subthemes with new data for theme refinement [19]. Coding co-occurred with data collection so that the interview script could be refined to explore early-identified open codes. Both coders open-coded the first several transcripts using these codes to build a codebook that included candidate themes and subthemes [19]. Once the coders determined that transcripts were not yielding new insights to contribute to the findings the team was generating (i.e., suggesting a high degree of saturation), the study team developed a timeline for ending data collection [20], recruiting an additional six participants to ensure gender balance. The coders then engaged in another round of comparison, where they applied the codebook to the remainder of the data using NVivo version 12. Once all transcripts were analyzed by both coders, the senior author (MT) acted as the validator, applying the codebook to four transcripts and comparing her discoveries to codebook themes. She also audited the codebook to suggest refinements. This process ensured that the codebook was an accurate representation of the synthesized data. The coders and validator discussed these and came to agreement on potential changes to the codebook. The coders then used NVivo queries to support or refute the validators' suggestions and incorporated these decisions into a final version of the codebook.

## 3 Results

Twenty-one older adults (*M*_*age*_ = 76.8) were included in this study (see Table 1). Purposive sampling resulted in a relatively equal distribution of men and women; about half reported their race as White. Most were highly educated. Care partners were not specifically recruited but were invited to engage with the My Health Priorities tool alongside participants if both desired. Thematic analysis generated three themes comprising eleven subthemes. Themes addressed: *user experience feedback* regarding accessing, using, and interacting with the My Health Priorities website, the *content of the health priorities identification process,* and the tool's *capacity to empower communication and decision making.*

**Table 1:** Participant Demographic Characteristics (N = 21).

Gender Identity (*n*, %)	
Man	10 (48)
Woman	11 (52)
Race (*n*, %)	
White	13 (61)
Black	1 (5)
Asian	2 (10)
Mixed-race	2 (10)
Other/ Did not disclose	3 (14)
Ethnicity (*n*, %)	
Hispanic/ Latinx	1 (5)
Non-Hispanic/ Latinx	20 (95)
Age (*M,* SD)	76.85 (4.77)
Highest degree earned	
High school/ GED	1 (5)
Associates/ 2-year degree	1 (5)
4-year degree	7 (37)
Graduate degree	11 (52)
Marital Status (*n*, %)	
Married	10 (48)
Widowed	5 (23)
Divorced	4 (19)
Single/ never married	2 (10)
Number of chronic conditions reported (*M, SD*)	3.85 (1.28)
Number of specialist providers regularly seen (*M, SD*)	2.42 (1.25)
Number of medications taken (*M, SD*)	4.75 (2.79)

*Note:* Participants categorized in “Other/ did not disclose” under Race did not identify with any racial group. In some cases, these participants identified based on their ethnic identity only; for example, one participant identified as ethnically Jewish. One participant did not disclose their highest degree earned. In some cases, when asked about the number of medications taken or chronic conditions, participants reported an estimated number or “more than” a certain number. For those cases, we recorded the number the participant provided, recognizing it is an approximate value, and used that approximate value to calculate averages and standard deviations. One participant did not disclose the number of medications they were taking.

### 3.1 User experience feedback

Users expressed differing experiences and satisfaction with the My Health Priorities tool. Some participants described that they found the tool to be just right for them as-is: “It was really simple. It's just a process of clicking on answers…. The language is easy… not complicated. And the steps were clear… it was user friendly.”. In other cases, participants described considerations in relation to two subthemes: their *appraisal of logistics and functionality* and their *appraisal of design*. Several participants initially described difficulty with or dislike for certain elements of the tool, but across the interview came to understand that their biggest barrier was their discomfort with engaging in a health priorities self-identification process (see *understanding the purpose of health priorities identification* subtheme below).

*Appraisal of logistics and functionality* of the My Health Priorities tool depended on technical and pragmatic preferences or needs. For example, while most participants reported being able to navigate the internet and understand user instructions, a small subset described relying on family members to navigate the web-based tool or reported difficulty navigating the tool because of limited experience with the internet. One participant described this difficulty:

The bar at the bottom includes “go back” and “next.” It takes up too much space… it covers some of the main material on the page. So someone would have to scroll down to see things on the page. And they might not realize that that's what they have to do.

Some participants found the closed-ended lists that are provided within the tool useful for moving through the process because they gave the participants tangible examples to consider, like one participant who described:

The choices given I thought were pretty good. And I was able to go through them and think about ‘em a little bit. And make a decision on it. That they provided enough examples that the person going through it could pick on of ‘em and just concentrate on that one… if somebody just came up to you blank and said, “All right. Tell me what *[your health goals]* are.” It's a little more difficult than seeing a list of things that are suggested, and then picking one of those.

Other participants, however, felt restrained by closed-ended lists and described preference for an open response format, as noted by one participant: “Not all the choices were relevant, and I was not always able to enter the information that is important to me. I do think that every time there's that option of “other,“ that somehow that didn't always seem enough.”

*Appraisal of design* involves participants' opinions of the aesthetics and scripting of the web-based tool. Because opinions ranged across users, suggestions for design improvement often contrasted each other. For example, some participants liked the inclusion of a fictional older adult because this feature allowed them to envision how they could respond to prompts throughout the process, as one participant noted, “It was really good to see an image of a person talking on the screen. It made it more like you were actually talking with someone.” However, others found the style of portraying the fictional older adult distracting or thought he represented an overly simplified version of the process:

While I was doing that, though, throughout the entire process, I wondered why the characters were so cartoonish. I didn't like that. Certainly my health is important and serious, and it just seemed to me there could be another vision image of—even like somewhat older people being sketched and really seen as people rather than the cartoony thing.”

Other *appraisals of design* were more unified; for example, multiple participants made suggestions for expanding the list of possible health goals.

### 3.2 The content of the health priorities identification process

The My Health Priorities Self-Identification tool takes users through a multi-step process, guiding them towards selecting one top health priority. Five subthemes described this process: *understanding the purpose of health priorities identification, illuminates what is important in life and health, clarifies current health goals, elicits one specific and relevant top health priority,* and *highlights the fluidity of health priorities and trade-offs.* Our analyses demonstrate that participants could describe benefits of, and movement through, each of these steps. Engagement with each step can be considered its own benefit of using the tool. Participants appreciated the flexibility of the tool, allowing them to move back and forth through the process as they reconsidered their most urgent or relevant top health priorities, as articulated by one participant:

One of the things that I absolutely don't want to have to do is go into the hospital. So I thought that was the most important thing, to stay out of the hospital. But as I went forward *[through the health priorities self-identification process]*, I realized that… *[it's]* not imminent… I don't have anything seriously wrong with me that's gonna get me in a hospital anytime soon… so it did… cause me to think about what is the real priority or what is the real important thing that's gonna prevent other things… it did make me… rethink what I thought were my priorities.

Engagement in the health priorities identification process relies on first *understanding the purpose of health priorities identification.* For many participants, the purpose of the tool was clear, and their understanding of the purpose aligned with the intentions of the creators of My Health Priorities. In some cases, participants struggled to understand the value of identifying a top health priority, or their expectations about the utility of this tool did not line up with the intentions of the team who created it, as described by one participant:

But I think my attention to health over the years has kept me from getting diabetes, and clinically obese, and all those other things… I don't know if I'm your best case for this… looking at this and saying, “Well, we're doing this decision thing where you hone in on one thing.” I was thinking, “Well, what if you got to pick a large number of things from a list?”

A few participants did not appreciate the benefits of a virtual self-identification process; for example, describing preference for social conversations about health priorities (versus self-directed identification), without recognizing that the tool could be a conversation facilitator. Though some participants conveyed difficulty *understanding the purpose of health priorities identification,* we note that this often did not preclude participants from finding parts the process beneficial.

Participants described that the My Health Priorities tool moved them through the process of health priorities self-identification by first *illuminating what is important in life and health*. Participants described that this tool shed light on the magnitude and urgency of care preferences they may have previously minimized, ignored, or overlooked. Participants also described how the tool acknowledged them as a ‘whole person,’ recognizing the myriad goals, values, and lived experiences that came into play when considering their health, as described by one participant, “It really got me to thinking beyond what the typical healthcare topics are. A lot of times you'll be focusing on your disease or your condition, and this was taking that step back and functioning more as a whole person.” Multiple participants articulated the value of doing this process individually, at home, to *illuminate what is important in life* even if they felt they had similar discussions with their clinicians, as noted by one participant:

I would encourage you to do it, even though you might think, “Oh, I've done this with my doctor.” Doing it on your own with some guidance, not in the doctor's office, it made me think of it from a different angle, and I learned something from it.

Participants next described that the tool *clarifies current health goals,* translating personal values to inform a range of specific health goals, as described by this participant, “You may be juggling a lot of different things in your life and with your health, and it helps you sort through that so that you can make some sense of what you should really focus on.” Participants noted that the process helped them focus on health goals that were important for the present, versus past or future goals, to ensure health decisions were aligned with maintaining current health.

Participants described that this tool helped them to narrow down multiple health goals and health problems to *elicit one specific and relevant top health priority.* Participants often noted that they felt their priorities were clear and relevant to them in the present, as described by this participant:

It was really nice for me to zero in on this particular topic or this particular idea of where I want to put effort… it was nice to learn that, hey, I mean, this has been a problem for me before. It's not that it didn't exist. I just didn't realize how much it was, and so it did help me to focus. I can say that's a good thing.

Some participants found that the tool helped them to clarify current health goals but found difficulty with determining just one top health priority; often, these participants described weighing several health goals equally. Finally, analyses demonstrated an additional benefit of engaging in this process, where the My Health Priorities tool *highlighted the fluidity of health priorities and trade-offs* required to pursue a health priority. For those participants who experienced this benefit, engaging recurrently in the priorities self-identification process was considered likely to be helpful for maintaining relevant health priorities as health conditions change over time, as noted by this participant:

And, to be honest with you, it might turn out a little bit differently, depending on the day, maybe you just got some new information, and then managing your health *[differently]* might become more important. So, I honestly think that this could be *[used]* more than once, and it would be interesting to… kind of chart it out and see how it tracks…

### 3.3 Capacity to empower communication and decision-making

Many participants felt that standard communication in clinical settings had room for improvement, as articulated by one participant, “Usually, *[clinicians]* don't really wanna talk to you about your ultimate goals or your health priorities or whatever. That's not usually the nature of my conversation with my doctors. Generally speaking, the doctors are mostly concerned with the physical.” This occurs, for example, when specialists were focused on specialty-specific short-term goals. One participant described:

Usually when I'm meeting with one of the healthcare professionals it's for a specific purpose, which is pretty much already pre-defined. If I'm going into the cardiologist for a, follow-up visit… they're not gonna ask me about what I think my long-term goals for life would be… We're gonna just talk about the reason for that visit. This website is certainly a more open-ended approach than what you would usually get in meeting with a clinician.

In response, participants described the benefits of the My Health Priorities tool across four sub themes: *“opens the door to conversations” with clinicians, enhances priorities-aligned clinical decision-making, aligns care across clinicians,* and *sows the seeds for conversations about care priorities with family and care partners*.

Participants described that the tool has the capacity to *“open the door to conversations” with clinicians* by ensuring conversations are bi-directional and emphasize the patient's health priorities, as described by this participant:

If my doctor had access to what I say here, then we would have things to talk about, perhaps, that I might not have raised. So, in that way it could change the nature of our conversation… it… has opened the door to a conversation that I might not otherwise have had.

Some participants felt they already had an open channel of communication with their clinicians, and as such were less likely to see possibilities that the My Health Priorities tool could improve conversations in clinic. One participant noted, “I have to say that my family practitioner, they do a very good job of asking a lot of questions any time I go there. I think they do have all this information in the computer already.”

Participants also described how the tool could *enhance priorities-aligned clinical decision-making.* By getting them in the mindset of participating in priorities-based decision-making. For example, one participant said:

And some of my medications, I didn't necessarily feel they were burdensome, but I probably should ask more questions about, “why have I been taking this for so long, and should I? Do I need to be taking it?” that kind of, like, gave me pause. I had to sit there and say, okay, I know what the medications are for, why I'm taking them.

Participants also felt this tool could mitigate feelings of intimidation in a clinical setting, as described by one participant, “A lot of the times the patient may feel intimidated by the doctor in that, ‘They know more than I do, and I need to follow what he does—or what he recommends’ as opposed to ask questions about why.” Finally, some participants described recognizing the value of this tool for *aligning care across clinicians.* With multiple specialists who each address different aspects of their health, many participants felt negative consequences of fragmented care. Some saw this tool, including the print-out summary page that they could bring to appointments, as an opportunity to get all clinicians on the same page about their health priorities and the trade-offs they were willing to make to address their priorities. One participant said, “Something like this might be helpful in opening up more of a dialogue… make all of the clinicians realize that it's a team effort.”

Participants also described how engaging with the My Health Priorities tool could *sow the seeds for conversations about care priorities with family and care partners*. Participants described how, when they informed family members or friends that they completed the priorities self-identification process, it reinforced for those family members or friends that the participant was serious about taking care of their health, as described by this participant, “Yesterday, when I finished the *[process]* and had a call from one of my neighbors, I explained it to him. He said to me, ‘Geez, I didn't know you were doing all the things you were doing to help yourself.’” Though care partners were not specifically instructed to participate in this study, some care partners had been invited by the participant to be involved. In one case, the care partner also noted benefits of the tool for illuminating the participants' health goals and trade-offs, which were previously unclear to them:

From a family point of view, I think this was really helpful in clarifying what her goals are. It sounds like in her mind she already knows what's important, but I really wasn't able to appreciate that as much… but I do think that this program did help clarify for the family member what is—what are trade-offs and what's the important thing for her.

A few participants appreciated this tool's application to family communication so much, they wished there was a step in the process that had them write out who their care partners were or who was included in their care support networks, as described by this participant:

Many times, we don't discuss our medical problems with our family members. On your website—I think you have not *[included]* who is taking care of me and I am taking care of whom in my family. So my primary healthcare provider *[can]* know our family background.

## 4 Discussion and conclusions

### 4.1 Discussion

Our findings illustrate the acceptability and ease of use of the My Health Priorities self-identification web-based tool in its current form. Although priorities identification is not currently standard practice in healthcare settings [3], many participants adopted the self-identification process with relative ease. In some cases, patients envisioned returning to the website to reconsider priorities over time. This process may be particularly important for older adults with MCCs whose abilities or health status change [21]. Findings also provide direction for addressing logistical and design concerns and point to opportunities to improve the reach of this priorities self-identification tool. Insights showcase the potential impact of this tool on clinical practice, including patient engagement in clinical discussions.

#### 4.1.1 Enhancing acceptability and reach: Website refinements and other considerations

While we incorporated user-centered design principles in the design of the My Health Priorities website [22], no tool can function to fit the exact needs or expectations of all users. For example, we noted some contrasting opinions of the website's content. A few users struggled with website navigation or language, despite our efforts to make these things accessible in the design phase. This feedback encompassed direct barriers to website use that offered guidance for refining this website [23]. Our findings also demonstrate that access to this tool may not be an ideal fit for initiating priorities identification in all users, including those users with limited technology capabilities.

Beyond logistical barriers, engagement with the website relies on interest in, and readiness to, begin the process of priorities self-identification. Participants seemed to have an easier time moving through the priorities identification process if they understood the purpose [12,24]. Instructions embedded on the website did describe the relevance and meaningfulness of completing the processes. These instructions may have been inadequate, or alternatively, instructions alone may be insufficient for some individuals to accept the usefulness of identifying their health priorities. Broader culture shifts to normalize health priorities identification processes may be critical for motivating a range of users to engage with priorities-identification tools. Clinician clarification about the value of a health priorities-identification process may be one opportunity to shift cultural norms and help users recognize the benefits of this tool.

Another indirect barrier to benefiting from health priorities identification was some users' impressions that their health care encounters already involved priorities-guided health decision making. While some clinicians may engage in true priorities-aligned discussions, it is possible that some participants conflated positive or friendly communication with priorities-aligned communication. Specifically, participants described that their clinicians used question-asking, empathy, or other rapport building approaches (e.g., one participant shared, *“I have a pretty good rapport with all of* [my clinicians]… *talk about anything and everything as well as just shoot the breeze sometimes…*”) to structure clinical discussions. While these approaches likely enhance patient satisfaction [25], they may not promote priorities-aligned health decision-making [26]. For example, one participant said, “*All the physicians that I have… we all have a very good rapport… some of them are more like friends.”* When asked as follow-up, “*Do you ever talk about what matters most? Have you ever been asked that by your healthcare team?”* the participant replied, “*No, I have not. I have to be honest.*”

Finally, in a few cases, participants described that the website prompted conversations with family members about health priorities, pointing to future applications of this website for dyadic priorities-aligned discussions between patients and care partners or other family members outside of clinical settings. Web-based tools can foster communication about family members' health preferences across care partner dyads [27], but few have been designed to elicit top health priorities. Our team has begun to explore the acceptability of the My Health Priorities tool as part of a dyadic intervention, particularly in populations of older adults living with dementia and MCCs [28]. Additional research should explore other applications of this tool for promoting dyadic patient-care partner health priority discussions.

#### 4.1.2 Limitations and future directions

Despite efforts to recruit a diverse sample, participants were, overall, highly educated older adults with access to computers and a modest number of reported chronic conditions. This sample is not representative of populations of older adults who are less educated or have limited computer or internet experience. We recognize the limitations posed by our sample. As socioeconomic status is a major predictor of health literacy, decision-making, and disparities [29], including in older adult population [30], decision-making tools should be tested within socially disadvantaged populations, including those individuals with less education or fewer technology resources. Community-based participatory research designs are one good approach for including a range of end users in clinical research and addressing resource access barriers [31], and should be used in future research on priorities self-identification tools.

Finally, all study interviews were conducted within a few days of participants completing the priorities self-identification process, so most participants had not yet had follow-up clinical encounters, some had not yet had chances to speak with family members or close others, and none had opportunities to use the website over time to re-consider their health priorities. Descriptions of this tool's benefits were constrained to what participants had already experienced or could confidently envision for future communication and decision-making contexts.

### 4.2 Innovation

The My Health Priorities web-based tool offers users a structured opportunity to identify health priorities outside of the clinical setting. This work is innovative in several ways. First, decision aides have been developed to support patient health decision-making in a variety of contexts where there is no objectively “correct” or “best” choice [32], but this is the first web-based tool to our knowledge designed specifically for older adults with MCCs to set health priorities. Unlike tools targeted towards discrete decisions about procedures or courses of treatment, this tool is grounded in determining what matters most to older adults with MCCs in the present [3], so that subsequent health decisions can be guided by users' top priorities. Second, our user-centered approach to developing this web-based tool allowed for iterative refinement and improvement. Findings from our research have informed modifications to this tool to improve international impact. Since launch, there have been over 12,000 users of the My Health Priorities tool across ten countries spanning five continents.

Finally, this tool provides patients with guidance for identifying their health priorities in advance of clinical encounters, improving their opportunity to steer clinical conversations towards priority-informed decision-making. Access to self-directed tools must be met with clinicians' readiness to engage in priorities-aligned discussions and decision-making; in other words, quality care for this population depends on both patients and clinicians working together to ensure decision-making is aligned with health priorities [9]. Our findings underscore the continued value of training clinicians to lead health priorities-directed decision-making for those patients who are least able to complete this process individually.

### 4.3 Conclusion

The My Health Priories self-directed website is a promising tool for older adults with MCCs to engage in a health priorities self-identification process on their own terms. With thoughtful refinements the website has the potential to serve a variety of benefits for many, albeit not all, older adults with MCCs who seek to align their health care with individualized priorities. Tools like this are vital for empowering patients to align clinical discussions with personal priorities.

## Funding

Dr. E. L. Mroz is supported by the 10.13039/100000049National Institute on Aging (NIA) Institutional Training Grant (T32AG019134). The study was supported in part by a grant from The John A. Hartford Foundation. Investigators received additional support and resources from the Claude D. Pepper Older Americans Independence Center at Yale University School of Medicine (#P30AG021342 NIH/NIA).

## Author contributions

MT and JE contributed to funding acquisition. MT, AN, and JE contributed to study conceptualization and supervision. KHB, EK and ELM contributed to data curation and formal analyses. MT contributed to validation. ELM and EK contributed to project administration. ELM lead writing of the original draft. All authors contributed to investigation and manuscript edits. All authors approved the final submission.

## CRediT authorship contribution statement

**Emily L. Mroz:** Writing – review & editing, Writing – original draft, Project administration, Methodology, Investigation, Formal analysis, Data curation. **Kizzy Hernandez-Bigos:** Writing – review & editing, Formal analysis, Data curation. **Jessica Esterson:** Writing – review & editing, Supervision, Funding acquisition, Conceptualization. **Eliza Kiwak:** Writing – review & editing, Project administration, Investigation, Formal analysis. **Aanand Naik:** Writing – review & editing, Supervision, Investigation, Conceptualization. **Mary E. Tinetti:** Writing – review & editing, Validation, Supervision, Investigation, Funding acquisition, Conceptualization.

## Declaration of Competing Interest

The authors have no competing interests to disclose.

## Figures and Tables

**Table:** 

• On a scale of 1 to 10, how do you rate your ability to use the internet to find information?
• First, I am going to ask about your experience with using a computer program on your own, the technical parts. Can you describe your experience with completing the entire program online? What worked well? What was more challenging?
• Now I would like to ask about your overall thoughts and reactions to the steps you worked through – identifying what matters most to you about your health and healthcare. How would you describe your experience identifying your life and health priorities; what was it like for you?
• The My Health Priorities website asks you to think about what gives your life meaning, purpose, joy, or satisfaction to guide selection of your health priorities. Can you tell me a bit about what thoughts came to mind when you considered these what gives your life meaning, purpose, joy, or satisfaction? What pieces of your own life came up for you when you reflected on these?
• How would you tell a friend what it was like to identify your health priorities? Would you recommend it to a friend/loved one? Why or why not?
• How does it compare with your typical encounters with health care professionals? How was it different from how you usually interact with your clinicians and providers?
• Which parts of the process do you think were particularly helpful for you to understand what matters most to you in your health and healthcare? Why?
• Which parts of the process do you think were particularly challenging? Why?
• How did this process help you and your caregiver/family communicate about and understand your health priorities, what matters most to you about your health and healthcare?
• How do you think this program will change the way you communicate and work with your primary care clinician or other clinicians?
• Is there anything you can suggest that would make it easier and better for people like you to complete this online program to identify their health priorities?
• Did you print the summary at the end of the program? If yes, did you share this with anyone?
• At this point, would you be comfortable sharing with me the one thing you selected as your health priority? Okay, and what was that one thing?

*Note:* This Table includes questions asked in the final version of the script, including questions that were added after data collection and analysis began, to probe further in places where thematic patterns arose early in data analysis.

**Table:** 

Area of Rigor	Description
Credibility	• Both supportive and discrepant cases used as central pillars of themes. • Analysis triangulation: coders used their different backgrounds to point out blind spots or challenge perspectives, and engaged in a validation process where the codebook was validated by an outside team members
Auditability	• Record retention: interview scripts, transcripts, codebook iterations, and memos about team decisions were saved and reviewed by the study team as needed.
Confirmability	• Use of NVivo to document systematic application of candidate themes and subthemes and accrual of exemplars • Candidate themes presented to team members before theme review
Transferability	• Coding and data collection occurred in tandem, allowing for modifications to the interview script and recruitment efforts to confirm or explore early-identified codes • Provision of sufficient descriptive data to support application to different populations • Publication of interview script
Dependability	• During data collection, regular touchpoints to discuss saturation and purposive sampling. • Comparison of transcripts across participants recruited from different sources to ensure codebook fit consistently with full sample.
